# Three‐Year Practice‐Based Clinical Trial on the Performance of a Self‐Adhesive Resin‐Based Bulk‐Fill Restorative

**DOI:** 10.1111/jerd.13468

**Published:** 2025-03-28

**Authors:** Andreas Rathke, Frank Pfefferkorn, Michael Kent McGuire, Rick H. Heard, Mikael Åström, Rainer Seemann

**Affiliations:** ^1^ Clinical Research Dentsply Sirona Konstanz Germany; ^2^ Faculty of Medicine University of Ulm Ulm Germany; ^3^ The McGuire Institute Houston Texas USA; ^4^ Department of Biostatistics StatCons Malmö Sweden; ^5^ Department of Restorative, Preventive and Pediatric Dentistry, ZMK Bern University of Bern Bern Switzerland

**Keywords:** bulk‐fill, clinical trial, composite hybrid, dual‐cure, practice‐based research, self‐adhesive, survival

## Abstract

**Objective:**

The prospective clinical study followed up on self‐adhesive resin‐based bulk‐fill restorations.

**Materials and Methods:**

Seven general dental practitioners from a practice‐based research network filled 60 cavities (20 Class I, 19 Class II, 21 Class V) in permanent vital teeth of 41 subjects with a self‐adhesive, dual‐curing composite hybrid (Surefil one). Modified USPHS criteria were evaluated at baseline and annually. Replacement or repair of the restoration was defined as failure. Data were analyzed using the Kaplan–Meier method and non‐parametrically (*p* < 0.05).

**Results:**

After 3 years (1118 ± 39 days), all 29 recalled teeth were rated as vital with no hypersensitivity. One tooth showed signs of cracking. One Class I and one Class II restoration failed due to a combined marginal gap and chipping. Two restorations showed a color mismatch. The remaining restorations were found to be in clinically acceptable condition and all Class II restorations in proximal contact maintained proper contact. With one restoration failure reported after 1 year, the total of three failures resulted in an annual failure rate of 3.94%.

**Conclusions:**

The self‐adhesive composite hybrid placed during daily routine showed acceptable results out to 3 years in load‐bearing Classes I and II as well as non‐retentive Class V cavities.

**Clinical Significance:**

Three‐year data confirmed the suitability of the novel self‐adhesive restorative material for stress‐bearing posterior restorations.

## 1 Introduction

Resin‐based composites have become the preferred filling material for direct restorations [1, 2]. Due to adequate mechanical properties such as flexural strength and wear resistance, they are also the main alternative to amalgam [2, 3]. Direct composite restorations have shown high clinical survival, with annual failure rate (AFR) values ranging from 0.08% to 6.3%, depending on various factors such as cavity class and size, tooth type and location, or follow‐up time [1, 2, 3, 4, 5, 6, 7]. The main reasons for failure were fractures and secondary caries, followed by esthetic concerns [1, 2, 3, 5, 6, 7, 8]. Conventionally, composites must be bonded to enamel and dentin with an adhesive and placed in single increments of 2 mm, which are light‐cured separately. Although the introduction of universal adhesives and bulk‐fill composites has simplified the restorative procedure in terms of bonding and placement of larger increments of up to 5 mm, the additional use of an adhesive is still required [9, 10, 11, 12]. Especially in cases where clinical procedures are difficult to perform, for example, when treating less accessible cavities or in special care patients who cannot tolerate prolonged treatments, prompt placement of restorative materials without custom retentive cavity preparation or adhesive steps is highly desirable [10, 13]. However, self‐adhesive materials such as glass ionomer cement (GIC), resin‐modified GIC, and self‐adhesive flowable composites are not always accepted for permanent use as load‐bearing restoration and/or have shown flexural strength below the acceptance level (80 MPa) defined in the ISO standard 4049 for polymer‐based posterior restorations [3, 10, 13, 14].

Continued research is focusing on self‐adhesive bulk‐fill restoratives that have higher load‐bearing capacity compared to their predecessors and are expected to perform better clinically in this respect. These novel materials are mainly developed based on a combination of functional adhesive monomer technologies and bulk‐fill composites through polymerizable acid polymers [15]. They are dual‐activated by a combination of light‐ and self‐curing, allowing bulk placement in one single step. A randomized controlled trial (RCT) found no difference in load‐bearing Class II restorations between an experimental self‐adhesive bulk‐fill resin‐based restorative (SABF; 3 M, St. Paul, MN, USA) and a bulk‐fill composite that was bonded with the corresponding universal adhesive in self‐etch mode [16]. After 3 years of clinical service, there was only one failure for both materials due to secondary caries, with a 96.6% survival rate. However, SABF was rated as less esthetic over the observed time periods. Another development in the same perspective, classified by the manufacturer as a self‐adhesive composite hybrid (Surefil one [SO]; Dentsply Sirona, Konstanz, Germany), was thoroughly verified in vitro and compared in prospective RCTs to conventionally bonded bulk‐fill composites. Except for one study that described a significant performance loss over 18 months with a 95% retention rate [17], all the SO restorations (100%) survived in Class I and/or II cavities after up to 2 years, and their success was comparable to conventionally bonded bulk‐fill composites [18, 19, 20]. In the latter study, esthetic shortcomings of SO in Class II restorations were reported, but no failures were observed [20]. In a practice‐based research network (PBRN) study which catalyzed the present study, only one SO restoration had been partially lost after 1 year, resulting in a 98% survival rate in Classes I, II, and V cavities [21]. Color changes were noted, but no other significant changes could be found. Despite these encouraging short‐term results, the evidence base is still limited for deciding to use self‐adhesive bulk‐fill restoratives confidently in dental practice. Observation periods of at least 3 years have been recommended for clinical assessment of direct restorative materials [22].

Therefore, the aim of the follow‐up investigation was to evaluate the clinical performance of SO restorations, which were placed during clinical routine in a PBRN after 3 years. The null hypothesis was that there are no changes in the clinical performance over the 3‐year observation period.

## 2 Materials and Methods

### 2.1 Study Design and Population

The practice‐based, prospective single‐arm trial was designed in accordance with good clinical practice, national guidelines, and regulations. The study protocol was approved by the Advarra Institutional Review Board in Columbia (MD, USA) in December 2018, with the number 00036511. The study involved five male and two female general dental practitioners (GDPs) who were members of a PBRN in the United States (The McGuire Institute, Houston, TX, USA) and had experience in the placement and evaluation of restorative materials. The dental practices were located in Houston and Missouri City (TX, USA).

The details regarding patient and tooth selection as well as the sample size have been published in the annual study [21]. In brief, the patients had to be older than 18 years, be in good general health, require at least one Class I, II, or V restoration in permanent teeth, and have no active periodontitis and rampant caries. Exclusion criteria were language barriers, severe medical complications or drug use, allergic history concerning methacrylate, lack of compliance, or pregnancy. Twenty male and 21 female patients (aged 21–78 years, mean 55.4 ± 14.3 years) who met the criteria were treated between January and March 2019 after giving written informed consent. All patients participated voluntarily with the right to withdraw from the study at any time. Twenty‐two participants received one restoration, and 19 of them received two restorations. The indications for restorations were caries lesions (*n* = 36) and replacement (*n* = 24).

### 2.2 Restorative Procedure

All seven GDPs were trained in the handling and application of the SO restorative prior to treatment, as previously described [21]. Each dental practice (*n* = 6) was asked to place 10 restorations, with two GDPs in one practice placing five restorations each. The material composition is shown in Table 1.

**TABLE 1:** Description of the two‐component restorative material according to the manufacturer.

Material	Manufacturer	Lot number	Composition
Self‐adhesive hybrid composite (SO)	Dentsply Sirona	1807004175	Powder: aluminum‐phosphor‐strontium‐sodium‐fluoro‐silicate glass, highly dispersed silicon dioxide, ytterbium fluoride, self‐cure initiator, camphorquinone Liquid: acrylic acid, polycarboxylic acid (MOPOS), bifunctional acrylate (BADEP), water Filler loading: 77 wt%, 58 vol%

Teeth to be restored reacted vital after testing and showed no (*n* = 59) or slight (*n* = 1) preoperative hypersensitivity. If indicated, local anesthesia was administered. Tooth shades were determined using the Vita classical shade guide (Vita Zahnfabrik, Bad Säckingen, Germany). The cavity size was determined by the original defect, that is, the defective restoration and/or caries lesion, and no additional retentions such as undercuts, dovetails, or bevels were prepared. The isthmus width was no more than two‐thirds of the intercuspal distance. Indirect pulp capping using calcium hydroxide (Dycal, Dentsply Sirona) was indicated in two preparations. Cases requiring direct pulp capping were excluded. The work field isolation was maintained with cotton rolls and suction in all but three cases, which were placed under rubber dam isolation. In Class II cavities, matrix systems with a dead soft, thin matrix band and accompanying wedges were applied. In Class V cavities, cervical matrices were used at the discretion of the GDPs. The cavities were cleaned with air‐water spray, leaving the cavity surface moist. The encapsulated SO in shade A3 was activated and mixed for 10 s in a capsule mixer (4000–6000 oscillations/min). The number of capsules applied depended on the cavity size. Using a capsule extruder, the mixed material was injected into the cavity in bulk while the capsule tip was gradually withdrawn. The material was adapted to the cavity margins and contoured using hand instruments. After self‐curing (6 min setting time from capsule activation) and optional light‐curing of the restorative surface for 20 s with a controlled light curing unit (≥ 800 mW/cm^2^), the occlusion was checked with articulating papers. Finishing and polishing was performed with silicon instruments (Enhance Finishing system, Dentsply Sirona) under water cooling. Patients were advised to contact their practice if they had any complaint or experienced pain.

### 2.3 Evaluation of the Restorations

Registration and case report forms were completed after placement of the restorations (baseline) and at the recall visits. All data management was carried out according to the Helsinki declaration. The restorations were monitored by the GDPs involved in the study and scored using the modified USPHS (United States Public Health Service) criteria [23, 24] shown in Table 2. The decision to intervene or not was taken by the GDP who placed the restoration. While the annual study reported the clinical results at baseline, after 3 months and 1 year [21], this study presented the results at baseline, two (753 ± 24 days) and three (1118 ± 39 days) years. For the evaluation, a visual and tactile examination was performed using magnifying glasses, dental mirrors, and explorers. At all recall visits, tooth vitality was checked and the patients were asked about persistent or new hypersensitivity. Intraoral photographs of the restorations were also taken at the recalls. Radiographs were performed if clinically indicated, for example for caries diagnosis or pain interpretation. Potential adverse events and side effects were recorded.

**TABLE 2:** Evaluation criteria and rating scale for the restorations.

Criteria	
Restoration quality	0 = Intact
	1 = Chipping
	2^a^ = Fracture
	3^a^ = Loss
Marginal quality	0 = Smooth
	1 = Step
	2^a^ = Gap
Tooth quality	0 = Sound
	1 = Cracking
	2^a^ = Fracture
Proximal contact	0 = Yes (Class II only)
	1^a^ = No (Class II only)
Caries	0 = No
	1^a^ = Yes
Vitality	0 = Yes
	1^a^ = No
Hypersensitivity	0 = No sensitivity is experienced at any time
	1 = Slight sensitivity is experienced occasionally but it is not uncomfortable
	2^a^ = Moderate sensitivity is experienced intermittently, and it is uncomfortable
	3^a^ = Severe discomfort is noted routinely with cold or pressure stimulation
Color match	0 = Perfect color match
	1 = Good color match
	2 = Slight color mismatch
	3^a^ = Obvious color mismatch
	4^a^ = Not at all satisfied
Color match (patient view)	0 = Perfect color match
	1 = Good color match
	2 = Slight color mismatch
	3^a^ = Obvious color mismatch
	4^a^ = Not at all satisfied

^a^ Unacceptable scores.

### 2.4 Statistical Analysis

Statistical unit was the tooth restored in the study. The need for repair or replacement was counted as failure. Data were analyzed using the software Stata (version 13.0, StataCorp LLC, College Station, TX, USA) and StatXact (version 12.0, Cytel Inc., Cambridge, MA, USA). The survival distribution was presented using the Kaplan–Meier method. Fisher's exact and Chi‐squared tests were used to test whether the distribution of the restorations differed between baseline and recall visits. The null hypothesis that there are no changes over time was tested by the non‐parametric Wilcoxon signed‐rank test for continuous variables and ordered categorical data and by means of the Binomial test for binary data. All *p*‐values were two‐sided. *p*‐values below 5% were considered statistically significant.

## 3 Results

**FIGURE 1:**
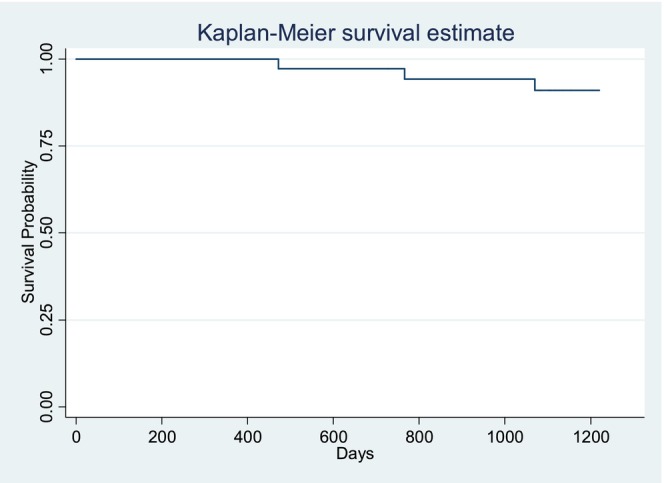
Kaplan–Meier survival plot for the SO restorations.

The recall rates at baseline and after 1, 2, and 3 years were 100%, 83%, 56%, and 49% respectively. The mean age of the patients at each recall was between 54 ± 14.5 and 56 ± 15.9 years, with a male/female ratio between 1.0 and 1.3. Dropout reasons were no further interest in study participation (*n* = 10), concerns related to the COVID‐19 pandemic (*n* = 4), moving away (*n* = 1), and losing track of patients (*n* = 6) because the practice terminated participation for reasons not related to the study. The distribution of the SO restorations is shown in Table 3. No significant differences were found in the distribution over time (*p* > 0.05). The Kaplan–Meier survival rate is illustrated in Figure 1. One Class I restoration in a lower premolar had to be replaced after 2 years (combined marginal gap and chipping) and one Class II restoration in a lower premolar was repaired after 3 years (combined marginal gap and chipping). With one failure (partial loss of a Class II restoration in an upper molar) reported in the annual study [21], a total of three failures resulted in an AFR of 3.94%.

**TABLE 3:** Distribution of SO restorations (absolute number (n) and percentage (%)) over time.

Patient	Baseline *n* (%)	Two years *n* (%)	Three years *n* (%)	Significance *p*‐value^a^	
1 restoration	22 (54%)	13 (57%)	11 (55%)	1.0000	1.0000
2 restorations	19 (46%)	10 (43%)	9 (45%)		
**Cavity**					
Class I	20 (33%)	13 (39%)	11 (38%)	0.3802	0.7804
Class II	19 (32%)	13 (39%)	10 (35%)		
Class V	21 (35%)	7 (22%)	8 (27%)		
**Cavity depth**					
> 2 mm RDT	32 (53%)	18 (55%)	16 (55%)	0.3390	0.4283
1–2 mm RDT	21 (35%)	14 (42%)	12 (41%)		
< 1 mm RDT	7 (12%)	1 (3%)	1 (4%)		
**Cavity width**					
1/3 ICD	25 (64%)	15 (58%)	14 (67%)	0.6143	1.000
2/3 ICD	14 (36%)	11 (42%)	7 (33%)		
**Tooth type**					
Canine	3 (5%)	1 (3%)	1 (4%)	0.8986	0.7451
Premolar	31 (52%)	17 (52%)	13 (45%)		
Molar	26 (43%)	15 (45%)	15 (51%)		
**Location**					
Maxilla	36 (60%)	22 (67%)	20 (69%)	0.6554	0.4867
Mandibula	24 (40%)	11 (33%)	9 (31%)		

*Note*: RDT remaining dentin thickness (estimated from radiographs), ICD intercuspal distance (Class I and II only). Results (baseline vs. 2 years left, baseline vs. 3 years right, 2 vs. 3 years not shown) were not significantly different (*p* > 0.05).

^a^ When comparing two categories, the Fishers exact test was used. When comparing three categories, the Chi‐squared test was used.

**FIGURE 2:**
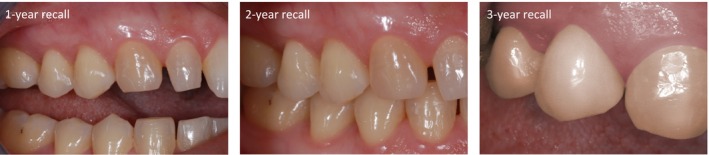
Representative images of the SO restoration evaluated. Class V restoration in the upper first premolar (tooth shade A3) at the annual recalls. A clinically acceptable phenotype could be observed after 3 years of clinical service (Courtesy of Dr. C. Alonso).

**FIGURE 3:**
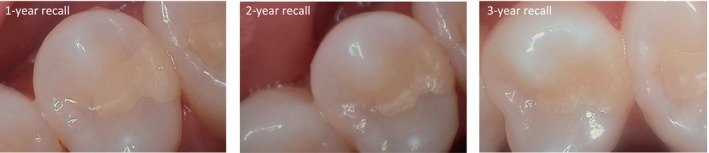
Representative images of the SO restoration evaluated. Class II restoration in the upper second premolar (tooth shade B1) at the annual recalls. A clinically acceptable phenotype could be observed after 3 years of clinical service (Courtesy of Dr. R. Hickerson).

**FIGURE 4:**
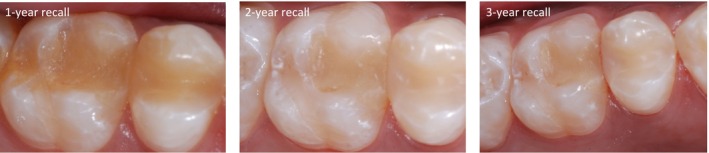
Representative images of the SO restorations evaluated. Class II restorations in the upper second premolar and first molar (tooth shades A1 and A2) at the annual recalls. Clinically acceptable phenotypes could be observed after 3 years. The restorations showed a typical observation of changes in color and translucency during clinical service (Courtesy of Dr. C. Alonso).

Details about the clinical performance of the SO restorations are presented in Table 4. Significant differences over time were found for the criteria “restoration quality,” “marginal quality,” and “color match” (*p* < 0.01). When comparing the results at 2 versus 3 years, the Wilcoxon signed‐rank test revealed similar outcomes for the evaluated criteria (*p* > 0.05). None of the restorations recalled after 3 years showed loss of vitality or hypersensitivity, and 83% (*n* = 24) of these restorations were clinically acceptable. The color match of SO restorations significantly improved over time (*p* < 0.0001). Two patients, including the one whose restoration was clinically unacceptable in color match, reported esthetic concerns. In both cases the tooth shade was A3. Illustrations of a representative sample of restorations are presented in Figures 2, 3, 4. No adverse events (other than failure rate) were observed in patients.

**TABLE 4:** Evaluation results of the SO restorations (absolute number [*n*] and percentage [%]) over time. The one‐year results of this study have been published previously [21].

Criteria		Baseline *n* (%)	Two years *n* (%)	Three years *n* (%)	Significance *p*‐value^a^	
Restoration quality	0	60 (100%)	32 (97%)	24 (83%)	0.3548	**0.0029**
	1	0	1 (3%)	5 (17%)		
	2	0	0	0		
	3	0	0	0		
Marginal quality	0	58 (97%)	26 (79%)	25 (86%)	**0.0088**	0.0854
	1	0	5 (15%)	2 (7%)		
	2	2 (3%)	2 (6%)	2 (7%)		
Tooth quality	0	59 (98%)	33 (100%)	28 (97%)	1.0000	1.0000
	1	1 (2%)	0	1 (3%)		
	2	0	0	0		
Proximal contact	0	18 (95%)	13 (100%)	10 (100)	1.0000	1.0000
	1	1 (5%)	0	0		
Caries	0	60 (100%)	33 (100%)	29 (100%)	1.0000	1.0000
	1	0	0	0		
Vitality	0	60 (100%)	33 (100%)	29 (100%)	1.0000	1.0000
	1	0	0	0		
Hypersensitivity	0	60 (100%)	33 (100%)	29 (100%)	1.0000	1.0000
	1	0	0	0		
	2	0	0	0		
	3	0	0	0		
Color match	0	3 (5%)	9 (27%)	9 (31%)	**0.0000**	**0.0000**
	1	6 (10%)	12 (37%)	4 (14%)		
	2	25 (42%)	10 (30%)	15 (52%)		
	3	17 (28%)	2 (6%)	0		
	4	9 (15%)	0	1 (3%)		
Color match (patient view)	0	—	21 (64%)	19 (65%)	—	—
	1	—	6 (18%)	8 (28%)		
	2	—	3 (9%)	0		
	3	—	1 (3%)	0		
	4	—	2 (6%)	2 (7%)		

*Note: p*‐values in bold show significant differences (Baseline vs. two years left, baseline vs. three years right). Two‐ versus 3‐year results were not significantly different (*p* > 0.05). —, Not determined.

^a^ Wilcoxon signed‐rank test and Binomial test.

## 4 Discussion

Most clinical studies on dental restorative materials are conducted at universities or other academic settings and not in dental practice where most treatments are performed. Clinical trials based on real‐world data from the use of materials in practice can therefore complement university‐based study designs [25]. This prospective clinical trial presented 3‐year data of a novel self‐adhesive resin‐based bulk‐fill restorative used by seven general dental practitioners (GDPs) working together with university staff in a US‐wide practice‐based research network (PBRN). According to the manufacturer, the evaluated self‐adhesive composite hybrid (SO) is indicated as permanent restorative for Classes I to V restorations. A total of three out of 60 Classes I, II, and V restorations failed during the study period, representing an AFR of 3.94% after 3 years. Two of the restoration failures were repaired instead of being completely replaced and their lifetime was defined as the period from baseline to the date of the clinical event ‘repair’. In daily practice, repair of partially defective restorations is an accepted treatment option and has been shown to prolong the survival of composite restorations [4, 5]. Unlike in this study, the lifetime of restorations where the repaired defect does not impair clinical function could also be defined as the period from baseline to the date of full replacement and considered as restorations in situ compared to event‐free restorations in situ [5].

Although the evaluation criteria were not as sensitive as required by university‐based studies and there was no comparison control, the use of the SO restorative in a PBRN team reduced the potential bias of a single‐center study and reflected the performance in daily practice. The null hypothesis tested in the study was rejected because significant changes in restoration quality, marginal integrity, and color match were observed over time. The reason for the significant downgrade in restoration quality was the increasing number of chipping fractures (17%); nevertheless, this was still within a clinically acceptable range. Neither bulk fractures nor secondary caries were found, indicating sufficient self‐adhesive and mechanical properties. The primary bonding mechanism of SO is a chemical (ionic) bond between the calcium ions of the hydroxyapatite and the carboxylic acid groups in both the hydrolytically stable MOPOS (modified polyacid system) monomer and in acrylic acid [15]. Self‐adhesion may also be modified by a micromechanical bond created by surface demineralization and hybridization or smear layer infiltration [21]. The results also indicated that the SO restorative was sufficiently bulk‐cured to withstand the occlusal forces. If the restorations had not been adequately cured in the deeper layers, bulk fractures into the poorly cured layer and failure of the adhesive bond would have been expected [11]. Fracture strength investigations have also shown the potential of both dual‐cured and self‐cured SO for use in posterior load‐bearing areas [26]. The absence of unresolved postoperative hypersensitivity can also be explained by the sufficient self‐adhesion and bulk‐curing. The results were consistent with clinical data from prospective randomized controlled trials (RCTs) showing that SO restorations were almost 100% successful in Class I and/or II cavities at their respective stage of evaluation (up to 2 years) [18, 19, 20]. However, as the present study was the first to evaluate SO restorations over a 3‐year period, the results were not readily comparable with those studies. A meta‐analysis of prospective studies on posterior composite restorations identified fractures, secondary caries, and marginal gaps as the main reasons for failure in the first 5 years [2]. In another meta‐analysis, fractures and secondary caries were found to be consistently important reasons for the failure of posterior composite restorations from the second year onwards [6].

After 2 and 3 years, two restorations failed due to the development of clinically unacceptable marginal gaps and chippings at the margins. Since 100% and 80% of the margins of Classes I and II restorations, respectively, are located in enamel, the significant loss of marginal quality can be partly explained by the relatively high pH of SO and the lack of selective enamel etching [27]. According to the manufacturer, the pH of SO is 2.1 directly after mixing and 3.2 after 6 min. The material then gradually becomes pH neutral. The inferior micro‐retentive etching pattern in enamel compared to 34%–37% phosphoric acid (pH 0.1–0.4) and/or chipping of resin flash or overhang extending over the unprepared enamel margin could contribute to the defects [21]. Equally important, the gap formation at baseline (3%) could have been caused by polymerization shrinkage, parallel orientation of enamel prisms at the cavity margins, or improper adaptation of the material to the cavity walls [3, 9]. Repairing or refurbishing composite restorations with marginal defects and superficial marginal discoloration was shown to increase their longevity [7]. However, most of the restorations showed marginal adhesion without reservation (86%) or clinically acceptable step formation (7%). The results were consistent with recent RCTs, which showed that the marginal quality of the SO restorative is comparable to that of bulk‐fill composites bonded with self‐etch adhesives [17, 18, 19, 20]. In two of these studies, however, the control composite performed significantly better in terms of marginal discoloration [17, 20].

The color match of SO restorations with the surrounding tooth structure improved significantly between baseline and the annual check‐ups. The color change could be attributed to increased translucency of the restorations compared to their more opaque or darker appearance at baseline. Recent RCTs compared the esthetic properties of the SO restorative with different light‐cured bulk‐fill composites. In two studies, the control composite performed significantly better than SO in terms of surface texture, color match, and translucency [17, 20], while another study found no difference in surface roughness and color match between the two materials [18]. One more RCT confirmed that a light‐cured bulk‐fill composite demonstrates better results with regard to surface luster, color match, and translucency than a self‐adhesive resin‐based bulk‐fill restorative (SABF) from the same manufacturer [16]. However, bulk‐fill composite shades tend to be more translucent than similar shades of conventional composites to increase the light transmission and curing depth during polymerization [12].

The sample size was well above the recommendations defined in the former American Dental Association acceptance program guidelines for posterior composite restorations, that is, at least 50 restorations at baseline with a maximum of two restorations per patient to accommodate study dropouts [24]. Despite several reminders, some patients failed to keep their appointments. Dropout rates are usually inevitable over time [28]. Several prospective studies suffered from a low recall rate [2, 8], for example, 24% after 2 years when evaluating GIC restorations [14], 44% after 3 years in the evaluation of a conventionally bonded bulk‐fill composite [11], or 33% after 6 years when evaluating compomer restorations [29]. A significant negative correlation between recall and failure rate was found [2]. In contrast, another meta‐analysis found a significant negative correlation between observation period and recall rate, but no correlation between recall and failure rate [8]. However, a low recall rate could cause bias in the sample available for evaluation. In the present study, the distribution of the SO restorations in terms of patient, cavity and tooth characterizations did not differ significantly between baseline and the other recalls. Furthermore, the sample size at last recall was sufficiently large to detect significant effects on the USPHS criteria “restoration quality,” “marginal quality,” and “color match” over time, allowing the null hypothesis to be rejected. However, a limitation could be that the study was potentially underpowered to detect other clinical effects due to the high dropout rate. Therefore, clinical investigations with increased sample size and prolonged observation time are needed to evaluate the novel self‐adhesive bulk‐fill resin‐based restorative.

## 5 Conclusion

The novel self‐adhesive bulk‐fill resin‐based restorative placed in a general dental practice setting showed clinically acceptable results out to 3 years in load‐bearing Classes I and II as well as non‐retentive Class V cavities. The results of this prospective study indicate the suitability of this self‐adhesive composite hybrid for stress‐bearing restorations.

## Ethics Statement

All procedures performed in studies involving human participants were in accordance with the ethical standards of the institutional research committee and with the 1964 Helsinki declaration and its later amendments or comparable ethical standards.

## Consent

Written informed consent was obtained from all individual participants included in the study.

## Conflicts of Interest

The authors declare no conflicts of interest.

## Data Availability

The data that support the findings of this study are available on request from the corresponding author. The data are not publicly available due to privacy or ethical restrictions.
